# Disentangling the body weight-bone mineral density association among breast cancer survivors: an examination of the independent roles of lean mass and fat mass

**DOI:** 10.1186/1471-2407-13-497

**Published:** 2013-10-25

**Authors:** Stephanie M George, Anne McTiernan, Adriana Villaseñor, Catherine M Alfano, Melinda L Irwin, Marian L Neuhouser, Richard N Baumgartner, Kathy B Baumgartner, Leslie Bernstein, Ashley W Smith, Rachel Ballard-Barbash

**Affiliations:** 1 Applied Research Program, Division of Cancer Control and Population Sciences, National Cancer Institute, Bethesda, MD 20892, USA; 2 Division of Public Health Sciences, Fred Hutchinson Cancer Research Center, Seattle WA, USA; 3 Moores UCSD Cancer Center, Cancer Prevention and Control Program, University of California, San Diego CA, USA; 4 Office of Cancer Survivorship, Division of Cancer Control and Population Sciences, National Cancer Institute, Bethesda MD, USA; 5 Division of Chronic Disease Epidemiology, MD Yale School of Public Health, New Haven CT, USA; 6 Department of Epidemiology and Population Health, University of Louisville, Louisville KY, USA; 7 Department of Population Sciences, Beckman Research Institute, City of Hope, Duarte CA, USA

**Keywords:** Body composition, Bone mineral density, Breast cancer survivor, Epidemiology, Bone loss

## Abstract

**Background:**

Bone mineral density (BMD) and lean mass (LM) may both decrease in breast cancer survivors, thereby increasing risk of falls and fractures. Research is needed to determine whether lean mass (LM) and fat mass (FM) independently relate to BMD in this patient group.

**Methods:**

The Health, Eating, Activity, and Lifestyle Study participants included 599 women, ages 29–87 years, diagnosed from 1995–1999 with stage 0-IIIA breast cancer, who underwent dual-energy X-ray absorptiometry scans approximately 6-months postdiagnosis. We calculated adjusted geometric means of total body BMD within quartiles (Q) of LM and FM. We also stratified LM-BMD associations by a fat mass index threshold that tracks with obesity (lower body fat: ≤12.9 kg/m^2^; higher body fat: >12.9 kg/m^2^) and stratified FM-BMD associations by appendicular lean mass index level corresponding with sarcopenia (non-sarcopenic: ≥ 5.45 kg/m^2^ and sarcopenic: < 5.45 kg/m^2^).

**Results:**

Higher LM (Q4 vs. Q1) was associated with higher total body BMD overall (1.12 g/cm^2^ vs. 1.07 g/cm^2^, *p*-trend < 0.0001), and among survivors with lower body fat (1.13 g/cm^2^ vs. 1.07 g/cm^2^, *p*-trend < 0.0001) and higher body fat (1.15 g/cm^2^ vs. 1.08 g/cm^2^, *p*-trend = 0.004). Higher FM (Q4 vs. Q1) was associated with higher total body BMD overall (1.12 g/cm^2^ vs. 1.07 g/cm^2^, *p*-trend < 0.0001) and among non-sarcopenic survivors (1.15 g/cm^2^ vs. 1.08 g/cm^2^, p < 0.0001), but the association was not significant among sarcopenic survivors (1.09 g/cm^2^ vs. 1.04 g/cm^2^, p-trend = 0.18).

**Conclusion:**

Among breast cancer survivors, higher LM and FM were independently related to higher total body BMD. Future exercise interventions to prevent bone loss among survivors should consider the potential relevance of increasing and preserving LM.

## Background

In the United States (US), over 2.5 million women are living with a personal history of breast cancer [1,2]. Age-related changes in body composition include a decrease in lean mass (LM), and loss and weakening of bone, leading to an increased risk of hip fractures and other fractures [3]. These changes are often accelerated by cancer and its treatment, including hormone therapies such as aromatase inhibitors [3]. Skeletal weakening is a particular concern for breast cancer survivors [4]. Compared to postmenopausal osteoporotic women without cancer, non-pathologic hip fractures in breast cancer survivors present at an earlier age and occur paradoxically at higher bone mineral density (BMD) [5]. Research has shown that after a diagnosis of breast cancer, survivors may have a 15% increased risk of falls and 55% increased risk of hip fracture [6], compared to postmenopausal women without cancer. The sequelae of fractures lead to many adverse events such as major surgery, increased morbidity and mortality, increased cost of disease management, and reduced quality of life [7].

Body weight has been proposed to be one of the best determinants of BMD [8]. Heavier women have a higher BMD because of the mechanical stress of weight on the skeleton [9]. However, for cancer survivors, being overweight or obese adversely affects quality of life, may worsen prognosis [10], and may increase the risk of chronic diseases, such as diabetes, hypertension, and coronary heart disease [11]. Further, obesity has been associated with greater risk of fall-related injury [12] and clinical fractures [13]. These latter associations may be due to difficulty maintaining postural stability [14] and/or diseases such as diabetes [14,15] which accompany obesity and are well-known to be associated with neuropathies and poor foot health. Because an increase in body fat over time has been shown to be common among women being treated for breast cancer [16-25], and because body weight does not necessarily track with increases in adipose tissue, [26] it is important to also understand the relationship of fat mass and BMD among women with breast cancer.

Among postmenopausal breast cancer survivors, targeted exercise training has been related to preservation of BMD [27,28]. Targeted exercise training can additionally help to prevent weight gain and concurrent losses in LM that result from cancer and its treatment, adding to its attractiveness as a lifestyle intervention of choice for survivors at risk for fractures and falls. However, the tailoring and testing of these targeted interventions is an area in progress [29], and given body weight’s strong relationship with BMD, it is useful to evaluate how the main components of weight, lean mass (LM) and fat mass (FM), relate to BMD so that interventions can be tailored as such to improve LM while reducing obesity. We explored this critical question in the Health, Eating, Activity, and Lifestyle (HEAL) Study of US women with early-stage breast cancer. We hypothesized that LM and FM would be independently related to total body BMD.

## Methods

### Study setting and participants

The HEAL study is a multi-ethnic prospective cohort study that has enrolled 1,183 women with first primary breast cancer drawn from Surveillance, Epidemiology, and End Results (SEER) population-based cancer registries in New Mexico, Western Washington State, and Los Angeles County. The study was designed to determine whether lifestyle, hormones, and other exposures affect breast cancer prognosis, and details of the study have been published, including physical activity levels of the population [30-32]. Around 6 months postdiagnosis, a subset of participants who were enrolled at New Mexico and Washington underwent a whole-body dual-energy X-ray absorptiometry (DXA) scan. To answer the study questions for this particular analysis, our sample included the women with this measure.

In New Mexico, we recruited 615 women aged 18 years or older, diagnosed with *in situ*, localized, and regional breast cancer between July 1996 and March 1999, and living in Bernalillo, Santa Fe, Sandoval, Valencia, or Taos counties. In Western Washington, we recruited 202 women between ages 40 and 64 years, diagnosed with *in situ* to regional breast cancer between September 1997 and September 1998, and living in King, Pierce, or Snohomish counties. The age range for the Washington patients was restricted due to other ongoing breast cancer studies. Patients were eligible if they were less than 12 months post-diagnosis. None of the patients used aromatase inhibitors; these drugs were not licensed for clinical practice at the time of their treatment. The study was approved by institutional review boards at all sites and informed consent was obtained from all participants. In Western Washington, a subset of 109 of the 202 participants were offered and participated in DXA scans; in New Mexico, all 615 participants were offered DXA scans and 499 participated. Of the 608 women who received DXA scans, we excluded one participant who was missing data on weight, and six participants missing data on current use of postmenopausal hormone therapy (estrogen or estrogen plus progestin). Our final sample included 599 women.

### Data collection

*DXA.* We used DXA to measure whole and regional body composition (New Mexico site: Lunar model DPX, Lunar Radiation Corporation, Madison, WI; Washington site: Hologic QDR 1500, Hologic, Inc., Bedford, MA). Data from the DXA scan were used to measure total body LM (g), FM (g), and BMD (g/cm^2^). Appendicular lean mass index (ALMI) was calculated as the sum of lean mass (fat-free, non-bone) in the arms and legs divided by height in m^2^[33]. We calculated fat mass index (FMI) as fat mass in kg / height in m^2^. DXA provides a highly reproducible and accurate measure for FM and LM and is a validated and accepted method for assessing body composition [34,35].

### Additional risk factors

Breast cancer stage at diagnosis was obtained from cancer registry records, and detailed information on treatment and surgical procedures was abstracted from cancer registry, physician, and hospital records. Height and weight were measured in-person. Information on age, race/ethnicity, smoking status, tamoxifen use, and postmenopausal hormone therapy use was determined via self-report using a standardized protocol. We categorized participants’ race/ethnicity as non-Hispanic white (n = 406); Hispanic (n = 94); and a combined category (n = 15) which included women who were Asian, American Indian, or “other” race. Menopausal status was determined via self-report and blood hormone levels of estradiol, estrone, and follicle-stimulating hormone.

### Statistical analysis

Log total body BMD values were regressed on quartiles (Q) of LM and FM in multivariate models, and beta scores were exponentiated and expressed as geometric means. We also performed the test for linear trend across categories of LM and FM, by assigning participants the median value of their categories and entering it as a continuous term in a regression model.

We also stratified the LM-BMD association by a FMI threshold for body that tracks with obesity status (lower body fat: <=12.9 kg/m^2^; higher body fat: >12.9 kg/m^2^) [36]. Similarly, we stratified the FM-BMD association by sarcopenia status using the ALMI cut point (non-sarcopenic: ≥ 5.45 kg/m^2^; sarcopenic: < 5.45 kg/m^2^) [37,38].

For model building, we identified risk factors that when added to exposure-outcome models acted as confounders (changing beta estimates by ≥ 10%) and were statistically significant (p < 0.05). Age and study site met these criteria for all relationships. LM-BMD models were adjusted for FMI and FM-BMD models were adjusted for ALMI. To enable comparability to the literature we also adjusted for menopausal status, cancer treatment, tamoxifen use, postmenopausal hormone therapy use, and race/ethnicity. Inclusion of these covariates in the models did not result in substantial changes to the beta values obtained in age-adjusted models. To further assure comparability with the extant literature focusing on skeletal health among postmenopausal women, we repeated analyses restricted to postmenopausal women.

All analyses were performed in SAS version 9.2 (Cary, NC). All tests were two-sided and statistical significance was set at p < 0.05.

## Results

At 6-months postdiagnosis, the mean age of participants was 57 (±11) years, and the majority of women were postmenopausal, non-Hispanic White, and not currently using postmenopausal hormone therapy (Table 1). At this time, about half of the women were taking tamoxifen, and none were using aromatase inhibitors.

**Table 1:** Demographic, clinical and lifestyle characteristics of 599 women in the health, eating, activity and lifestyle study, 6 months postdiagnosis

	**Mean**	**SD**	**N**	**%**
Age (yrs)	57.5	11.4		
BMI (kg/m^2^)	26.1	5.3		
Lean mass (kg)	39.7	5.6		
Appendicular lean mass (kg)	16.6	2.6		
Fat mass (kg)	26.4	10.2		
Bone mineral density (g/cm^2^)	1.1	0.1		
Obesity status by FMI levels				
No (FMI < =12.9 kg/m^2^)			474	79
Yes (FMI > 12.9 kg/m^2^)			125	21
Sarcopenia status				
No (ALM index > =5.45 kg/m^2^)			515	86
Yes (ALM index < 5.45 kg/m^2^)			84	14
Study site				
Western Washington			103	17
New Mexico			496	83
Race/ethnicity				
Non-Hispanic white			464	77
Hispanic			120	20
Asian, American Indian, Other			15	3
Menopausal status				
Premenopausal			201	34
Postmenopausal			379	63
Unknown			19	3
Current postmenopausal hormone therapy use				
No			585	98
Yes			14	2
Current tamoxifen use				
No			308	51
Yes			291	49
Treatment beyond surgery				
No chemotherapy or radiation			195	33
Radiation only			250	42
Chemotherapy only			37	6
Radiation + chemotherapy			117	20

As shown in Table 2, higher vs. lower (Q4 vs. Q1) LM was associated with higher BMD (1.12 g/cm^2^ vs. 1.07 g/cm^2^, *p*-trend < 0.0001) and higher vs. lower (Q4 vs. Q1) FM was associated with higher BMD (1.12 g/cm^2^ vs. 1.07 g/cm^2^, *p*-trend < 0.0001).

**Table 2:** Multivariate adjusted geometric means and 95% confidence intervals of bone mineral density (BMD) by quartiles of lean mass and fat mass

	**Quartile (Q)**	**N**	**Geometric mean (95% CI) of BMD (g/cm**^**2**^**)**
Lean Mass (kg)^1^			
	Q1 (26–36)	149	1.07 (1.04, 1.10)
	Q2 (36–39)	150	1.09 (1.06, 1.13)
	Q3 (39–43)	150	1.10 (1.07, 1.13)
	Q4 (43–70)	150	1.12 (1.08, 1.15)
	*p*-trend		< 0.0001
Fat Mass (kg)^2^			
	Q1 (3–19)	149	1.07 (1.04, 1.10)
	Q2 (19–25)	150	1.09 (1.06, 1.12)
	Q3 (25–32)	150	1.09 (1.06, 1.12)
	Q4 (32–68)	150	1.12 (1.09, 1.16)
			< 0.0001

^1^Adjusted for age, menopausal status, postmenopausal hormone therapy use, treatment at diagnosis, tamoxifen use, race/ethnicity, study site, and *fat mass index.*

^2^Adjusted for age, menopausal status, postmenopausal hormone therapy use, treatment at diagnosis, tamoxifen use, race/ethnicity, study site, and *appendicular lean mass index.*

As shown in Table 3, in stratified analyses by body fatness and sarcopenia, higher vs. lower LM was associated with BMD both in survivors with lower body fat (1.13 g/cm^2^ vs. 1.07 g/c, *p*-trend < 0.0001) and higher body fat (1.15 g/cm^2^ vs. 1.08 g/cm^2^, *p*-trend = 0.004). Higher vs. lower FM was associated with BMD among non-sarcopenic women (1.15 g/cm^2^ vs. 1.08 g/cm^2^, p < 0.0001); however, among women with sarcopenia, the FM-BMD relationship was in a similar direction and of comparable magnitude but was not statistically significant (1.09 g/cm^2^ vs. 1.04 g/cm^2^, p-trend = 0.18). When we repeated analyses among postmenopausal women, results were similar (data not shown).

**Table 3:** Multivariate adjusted geometric means and 95% confidence intervals of bone mineral density (BMD) by quartiles of lean mass and fat mass by body fatness and sarcopenia status

	**Women with fat mass index < = 12.9 kg/m**^**2 **^**(n = 474)**			**Women with fat mass index > 12.9 kg/m**^**2 **^**(n = 125)**		
	**Quartile (Q)**	**N**	**Geometric mean (95% CI) of BMD (g/cm**^**2**^**)**	**Quartile (Q)**	**N**	**Geometric mean (95% CI) of BMD (g/cm**^**2**^**)**
Lean Mass (kg)						
	Q1 (26–35)	118	1.07 (1.04, 1.11)	Q1 (31–38)	31	1.08 (1.04, 1.12)
	Q2 (35–38)	119	1.09 (1.06, 1.13)	Q2 (38–43)	31	1.08 (1.04, 1.12)
	Q3 (38–41)	119	1.10 (1.06, 1.13)	Q3 (43–47)	32	1.13 (1.09, 1.17)
	Q4 (41–57)	118	1.13 (1.09, 1.17)	Q4 (47–70)	31	1.15 (1.11, 1.19)
	*p*-trend		< 0.0001	*p*-trend		0.004
	**Women without sarcopenia (n = 515)**^**2**^			**Women with sarcopenia (n = 84)**^**2**^		
Fat Mass (kg)						
	Q1 (3–20)	128	1.08 (1.05, 1.12)	Q1 (6–17)	21	1.04 (0.97, 1.12)
	Q2 (20–26)	129	1.11 (1.07, 1.14)	Q2 (17–20)	21	1.07 (0.97, 1.17)
	Q3 (26–33)	129	1.11 (1.07, 1.14)	Q3 (20–24)	21	1.08 (0.99, 1.18)
	Q4 (33–68)	129	1.15 (1.11, 1.19)	Q4 (24–34)	21	1.09 (0.99, 1.20)
	p-trend		< 0.0001	p-trend		0.18

^1^Adjusted for age, menopausal status, postmenopausal hormone therapy use, treatment at diagnosis, tamoxifen use, race/ethnicity, study site.

^2^Sarcopenia status determined by appendicular lean mass index; without sarcopenia: ≥ 5.45 kg/m^2^, sarcopenic: < 5.45 kg/m^2^.

In univariate models, LM also explained more variance in BMD among those who were not obese (10%) than among those who were obese (3%), and FM explained more variance in BMD among those who were not sarcopenic (3%) than among those who were sarcopenic (0.005%) (data not shown). In this study with its wide age range, age explained more variance (8-18%) in BMD in all univariate models than other variables (data not shown).

## Discussion

Our results demonstrate the independent association of LM and total body BMD among breast cancer survivors, after controlling for relevant confounders, and confirmed the role of FM in relation to total body BMD. This finding showing the importance LM in relation to BMD is biologically plausible, because dynamic rather than static loads promote bone formation and retention [39,40], and adipose tissue (FM) predominantly applies a static load on the bone; in contrast, muscle tissue (LM) exerts a dynamic strain on bone [15].

Rates of true bone loss among postmenopausal women are reported to be approximately 3% per year [41]. The cross-sectional differences in total body BMD observed in our study for those in the highest vs. lowest quartiles of LM (5%) and FM (5%) suggest clinical relevance. However, since this is the first investigation on this topic among breast cancer survivors, these observations require validation. Among survivors, some physical activity interventions have promise in affecting both LM and BMD. Among cancer survivors, postdiagnosis weight-bearing physical activity and resistance training have been shown to increase LM [29], and among postmenopausal breast cancer survivors at risk for bone loss, postdiagnosis strength/weight training has been shown to prevent loss of BMD [27,42]. More comprehensive and longitudinal research is needed to understand how and the extent to which different types and doses of physical activity affect both LM and BMD.

Key strengths of this study include our large group of breast cancer survivors ascertained through US population-based cancer registries and the use of DXA to assess body composition. This study also has some limitations. Although this study also included a fairly diverse sample of US Non-Hispanic White and Hispanic women, the generalizability of our findings may be limited, and it will be important to extend this research to populations from other cultures and race/ethnicities where some research suggests that the association between body composition and BMD may vary from that observed in white populations. In addition, our cohort only included women with breast cancer, so we are unable to compare associations observed in survivors with those observed in women without cancer. Many of the body composition changes experienced by breast cancer patients happen during and after treatment, and the DXA measures collected in our study occurred at one point in the time, approximately 6 months post-diagnosis. Because our study only had a measure of total body BMD, we were not able to examine associations with spine or hip BMD, which would be most clinically relevant. Our measure of total fat mass did not allow us to break down the associations by types of adipose tissue, such as visceral fat. Future studies with multiple, comprehensive measurements of body composition throughout the cancer treatment process could identify critical periods of rapid bone loss.

We did not have data on recent bisphosphonate use, osteoporosis, or comorbidities at the time of the 6-months post-diagnosis assessment, so we were unable to control for these factors. In addition, the cohort accrued participants before the widespread use of aromatase inhibitors; therefore our study could not address associations among women treated with these medications. Lastly, we had very few patients with sarcopenia (n = 84), which limited our ability to draw conclusions about the association of FM-BMD in this group, or the heterogeneity by sarcopenia status.

## Conclusion

Within this large study of early-stage breast cancer survivors, we found that higher LM was independently related to higher total body BMD. Given breast cancer survivors’ increased risk for falls and fractures, replication of our results in other cohort studies could determine whether preservation of LM results in meaningful reductions in bone loss among breast cancer patients. Future exercise interventions to prevent bone loss among survivors should consider the potential relevance of increasing and preserving LM.

## Abbreviations

ALMI: Appendicular lean mass index; BMD: Bone mineral density; CI: Confidence interval; DXA: Dual-energy X-ray absorptiometry; FM: Fat mass; FMI: Fat mass index; HEAL: Health, Eating, Activity, and Lifestyle Study; LM: Lean mass; Q: Quartile.

## Competing interests

The authors declare that they have no competing interests.

## Authors’ contributions

Substantial contributions to the conception and design: SMG, RBB, AV, AM, MLN, and RNB. Acquisition of data RBB, AM, MLN, AV, RNB, KBB, LB. Interpretation of the data: SMG, AM, AV, CMA, MLI, MLN, RNB, KBB, LB, AWS, and RBB. Drafting of the manuscript: SMG, AM, AV, CMA, MLI, MLN, RNB, KBB, LB, AWS, and RBB. Critical revisions: SMG and RBB. Final approval of the version to be published: SMG, AM, AV, CMA, MLI, MLN, RNB, KBB, LB, AWS, and RBB.

## Pre-publication history

The pre-publication history for this paper can be accessed here:

http://www.biomedcentral.com/1471-2407/13/497/prepub
